# CONTRALATERAL PATELLAR TENDON AUTOGRAFT IN ANTERIOR CRUCIATE LIGAMENT RECONSTRUCTION

**DOI:** 10.1590/1413-785220182602185594

**Published:** 2018

**Authors:** PAULO LOBO, EUGÊNIO DOS SANTOS, JOSÉ HUMBERTO DE SOUZA BORGES, LUANA JUNQUEIRA RESENDE VOLPE DIAS, RONNY DE SOUZA MACHADO, ANDERSON FREITAS

**Affiliations:** 1. IPE-HOME, Hospital Ortopédico e Medicina Especializada, Brasília, DF, Brazil.

**Keywords:** Anterior cruciate ligament, Patellar ligament, Knee joint, Tendons/transplantation, Ligamento cruzado anterior, Ligamento patelar, Articulação do joelho, Tendões/transplante.

## Abstract

**Objective:**

To conduct a systematic review of literature about the use of contralateral patellar tendon autograft in anterior cruciate ligament reconstructions and present the results.

**Methods:**

The LILACS, MEDLINE, Cochrane, PubMed, Scielo and Google Scholar databases were searched without date restrictions for the keywords “anterior cruciate ligament reconstruction” combined with “contralateral” in the article title. After the studies were identified, two independent evaluators collected the qualitative characteristics of the studies and classified them according to clinical outcomes of these grafts as positive, neutral, or negative.

**Results:**

A total of 755 articles were found initially, and after detailed evaluation of all references, followed by a screening process and assessment of quality, a total of 11 studies were determined to be eligible for inclusion in this systematic review. Of these, 72.72% were level II studies, the most common level of evidence among the results. Positive results for this type of graft were found in 63.63% of the studies.

**Conclusion:**

Based on the literature review, most of the included articles (63.63%) presented positive results for the use of contralateral patellar tendon grafts. Level of Evidence III; Systematic review.

## RESUMO

**Objetivo:**

Realizar uma revisão sistemática da literatura abordando o uso do tendão patelar contralateral como enxerto nas reconstruções do ligamento cruzado anterior e apresentar seus resultados.

**Métodos:**

Foi realizado um levantamento nas bases de dados LILACS, MEDLINE, Cochrane, PubMed, Scielo e Google acadêmico sem restrição de data e utilizando como descritores as palavras: “anterior cruciate ligament reconstruction” combinadas com “contralateral” no título do artigo. Após a identificação dos estudos, dois avaliadores independentes coletaram características qualitativas dos estudos, as quais foram classificadas de acordo com os resultados clínicos como positivas, indiferentes e negativas para o uso desse enxerto.

**Resultados:**

Foram encontrados, inicialmente 755 artigos e após uma avaliação detalhada de todas as referências, seguida de processo de triagem e avaliação da qualidade, um total de 11 estudos foram determinados como elegíveis para inclusão nesta revisão sistemática, sendo que 72,72% deles apresentaram nível de evidência II, sendo este o maior nível encontrado; 63,63% apresentaram resultado positivo para esse tipo de enxerto.

**Conclusão:**

Com base na revisão da literatura, a maioria dos artigos avaliados (63,63%) apresenta resultados positivos para o uso do enxerto do tendão patelar contralateral. Nível de Evidência III; Revisão sistemática.

## INTRODUCTION

The anterior cruciate ligament (ACL) plays a very important role in knee biomechanics; it is the primary stabilizer against anterior tibial translation, and acts as a secondary stabilizer in excessive internal rotation and in stress in valgus and varus. Because of the high incidence of ACL injuries in the population, this ligament has been the subject of many contemporary studies. Considering the instability ACL injuries cause, and potential comorbidities resulting from ACL rupture (such as meniscal and chondral injury and possibly early osteoarthrosis), the recommended treatment is surgical and involves ligament reconstruction.^1^ ^,^ ^2^

An improved surgical technique for reconstructing this ligament has made it less invasive, and when combined with early rehabilitation leads to joint stability and decreases the time patients need to return to their normal activities (not only professional sports, but particularly to work in ordinary patients), which has greatly increased the number of surgical reconstructions in recent years.^3^

Despite the frequency of ACL reconstruction surgery, extensive experience among orthopedic surgeons, and increasing advancement in surgical techniques, the choice of graft to be used in ligament reconstruction remains a main topic of debate in the literature.^2^ ^,^ ^4^

Currently, several graft sources have been shown effective in ACL reconstruction; the choice of the ideal graft is tailored to the patient profile and the injury, and also is affected by the surgeon’s personal experience. Grafts derived from the quadriceps tendon and the flexors have emerged as an option for patellar grafting, following studies on anterior knee pain and morbidity of the donor site.^4^

With increasing numbers of revision procedures after ACL reconstruction surgery, there was a need for grafts from other sites such as the contralateral patellar tendon. Clinical observations by surgeons choosing this new site produced results equaling or even exceeding those of the primary surgery, raising the possibility that this site could be a good donor candidate for primary reconstructions.^5^ ^-^ ^7^

Because of the importance and scarcity of scientific studies on the use of the contralateral patellar tendon as a graft in reconstructing the ACL, the authors conducted this review, which is principally intended to present the results of studies that used this graft in primary reconstructions of the ACL of the knee.

## MATERIALS AND METHODS

A systematic review of the literature in the LILACS, MEDLINE, Cochrane, Scielo, PubMed and Google Scholar databases was conducted. The search was not limited to any date range because of the need for a historical delineation. The text keywords we used were: “anterior cruciate ligament reconstruction” combined with “contralateral”, and we always searched for these terms in the article title.

The selection criteria for studies included in this review were:

- articles written in English or Portuguese;
- theoretical studies matching the objectives of this analysis;
- cross-sectional studies comparing graft types including contralateral patellar tendon grafts;
- correlation studies involving the use of the contralateral patellar tendon as a graft;
- controlled clinical trials verifying outcomes from the use of the contralateral patellar tendon as a graft.

Exclusion criteria were:

- articles in languages other than English or Portuguese;
- articles with deficiencies in their methodology section, principally with regard to selection and sample power, as well as materials used;
- studies which did not involve the use of the contralateral patellar tendon as a graft;
- case reports or case series involving fewer than five patients;
- letters to the editor or expert opinions.

Data were collected from May 2017 to August 2017. The main author of this study evaluated all the articles and applied the inclusion and exclusion criteria. Each relevant study was obtained and reviewed in its entirety. The studies were identified electronically, based on the abstracts and full texts in the databases. After the studies were identified, two evaluators independently collected the qualitative characteristics and results of the studies, grouping them as positive, negative, or neutral. Any doubts related to selecting the articles or their results were resolved by consensus between the two researchers. When questions remained, a third reviewer was consulted to achieve a majority opinion.

This level of evidence has been determined in accordance with the Oxford Levels of Evidence Classification System produced by the Oxford Center for Evidence-Based Medicine.^8^

## RESULTS

Searching for the terms “anterior cruciate ligament reconstruction” combined with “contralateral” in the article titles, we initially identified 755 articles from the electronic databases consulted. (Table 1)

**Table 1:** Number of articles found on May 1, 2017 according to database.

Database	Number of articles
Pubmed	22
Bireme	557
Lilacs	26
Google Scholar	68
Cochrane	67
Scielo	15
Total	755

**Figure 1:**
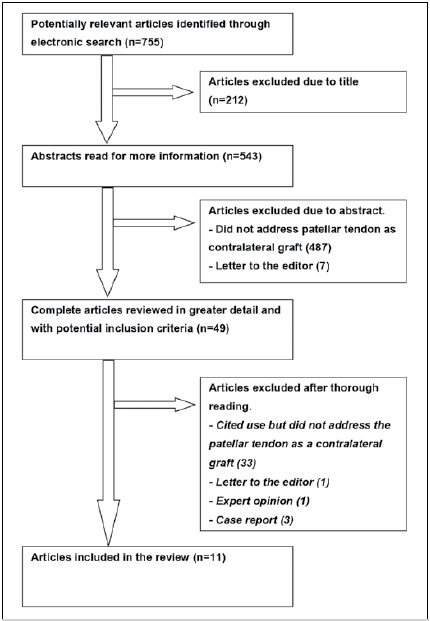
Flowchart of study selection.

After detailed evaluation of all references followed by a screening process and quality assessment, a total of 11 studies were determined eligible for inclusion in this systematic review, as shown in Figure 1.

Table 2 presents the lead author, year of publication, article title, most important findings presented in the article, level of evidence, and clinical outcome. The highest level of evidence found was level II, which was also the most frequent, with 72.72% of studies (8). Level III was found in 27.28% studies (3); no articles were found with level I, and studies with levels IV and V were excluded from this review. This review also found that 63.63% of studies (7) presented positive results for the use of the contralateral patellar tendon as a graft, 27.27% studies (3) contained neutral results, and 9.09% of studies (1) presented negative results for use of this graft.

**Table 2:** Reference, title, conclusion, level of evidence, and results for each study selected for bibiliographic review.

Reference	Title	Conclusion	Level of Evidence	Result
Rubinstein et al.^5^, 1994.	Isolated autogenous bone-patellar tendon-bone graft site morbidity	The morbidity from collecting contralateral patellar tendon grafts seems to be of short duration and largely reversible.	2C	P
Uribe et al.^6^, 1996.	Revision anterior cruciate ligament surgery: experience from Miami.	Collection of the contralateral patellar tendon was not found to produce adverse effects in the long term. Learning several anterior cruciate ligament reconstruction techniques and avoiding tunnels and pre-existing implants facilitate surgical revision. Correct placement of the graft and addressing secondary constraints are essential for a successful revision surgery.	2C	I
Kartus et al.^9^, 1998.	Ipsi- or contralateral patellar tendon graft in anterior cruciate ligament revision surgery. A comparison of two methods.	Recovery after anterior cruciate ligament reconstruction using the ipsilateral patellar tendon resulted in lower functional scores and a greater rate of complications than revision with the contralateral patellar tendon.	2B.	P
Bruck et al.^10^,1998.	Morbidity after contralateral transplantation of the patellar ligament for cruciate ligament replacement	There was no radiographic evidence of low patella. Use of contralateral patellar tendon graft for anterior cruciate ligament reconstruction does not involve severe morbidity.	2C	P
Shelbourne et al.^11^, 2000.	Primary anterior cruciate ligament reconstruction using the contralateral autogenous patellar tendon.	The contralateral patellar tendon may be used to restore range of motion and muscle strength earlier than ipsilateral patellar tendon graft. Patients may also return to full capacity in sports more quickly without compromising maximum stability.	2A	P
Shelbourne et al.^12^, 2005.	Contralateral patellar tendon and the Shelbourne experience Part 2. Results of Revision Anterior Cruciate Ligament Reconstruction	The objectives of ACL reconstruction revision should be similar to those of primary surgery, restoring stability, movement, and function. Suggests using the contralateral patellar tendon graft and mini-arthrotomy technique Provides similar results to the primary procedure and has consequently become the method of choice for primary ACL reconstruction.	2B	P
Zink et al.^13^, 2005.	Gender comparison of knee strength recovery following ACL reconstruction with contralateral patellar tendon graft.	Since strength recovery after surgery is not identical in men and women, specific rehabilitation protocols for each sex may be justified.	2C	I
Mastrokalos et al.^14^, 2005.	Donor site morbidity and return to the preinjury activity level after anterior cruciate ligament reconstruction using ipsilateral and contralateral patellar tendon autograft: a retrospective, nonrandomized study	Contralateral patellar tendon graft appears to offer no advantages over ipsilateral grafts, because all symptoms related to morbidity of the donor site are transferred to the healthy knee, and patients do not return to activity earlier.	2A	N
Benner et al.^15^, 2011.	Infections and patellar tendon ruptures after anterior cruciate ligament reconstruction A comparison of ipsilateral and contralateral patellar tendon autografts	There were no significant differences in the incidence of infection or patellar tendon rupture between the ipsilateral and contralateral groups. Patients with complications after ACL reconstruction using an autogenous patellar tendon graft may have less difficulty achieving complete knee movement when the graft is collected from the contralateral knee.	3A	P
Dauty et al.^16^, 2014.	Muscular isokinetic strength recovery after knee anterior cruciate ligament reconstruction revision: Preliminary study	Deficits in isokinetic muscle strength after ACL revision seem similar to those observed after primary ACL reconstruction using the same surgical technique and patellar, ipsilateral ischiotibial, and contralateral patellar grafts.	3A	I
Shelbourne et al.^17^, 2015.	Anterior cruciate ligament reconstruction with contralateral autogenous patellar tendon graft: evaluation of donor site strength and subjective results.	After ACL reconstruction using a contralateral patellar graft, patients can achieve symmetrical force in the legs without adverse subjective symptoms after graft collection. Additionally, there may be more return with a contralateral graft than with an ipsilateral graft.	3A	P

## DISCUSSION

Over the last 40 years, the therapeutic approach to ACL injury has undergone significant alterations, returning to the 1939 technique which used the patellar tendon to replace the ruptured ACL. This return to the old technique was only possible through advances in anatomy and biomechanics, along with arthroscopy using new tools and fixation techniques which provide earlier rehabilitation and better results.^18^

Besides good fixation, the current goal is anatomical reconstruction of the ACL in order to reestablish the structural and biomechanical properties of the knee, mainly with regard to rotational instability. The items that contribute to satisfactory progress after this procedure are adequate choice of surgical technique for each patient, the condition of the secondary restrictors (meniscus and ligaments), post-operative analgesia, and early and safe rehabilitation. Improvements and innovations in ACL reconstruction techniques have produced satisfactory results for instability control and early return to sports.^3^ ^,^ ^18^

Since it is a surgical procedure, ACL reconstruction presents complications inherent to any intervention, such as healing problems, deep venous thrombosis, infection, and hemorrhage. However, there are specific complications arising from the use of different graft types.^1^ ^,^ ^2^

During the study period, several types of grafts were used: autologous, allografts, and synthetic. Currently, the tendency is to use a strong biological graft; autologous grafts from the patellar and ischiotibial tendons (semitendinosus and gracilis tendons) are the most frequently discussed. One of the complications which have been most widely studied with regard to the use of patellar tendon graft is patellar fracture. When the ischiotibial tendons are used, a possible complication is that patients may present some deficit of knee flexion in the donor knee.^11^ ^,^ ^12^

Among the most frequent complications of ACL reconstruction, pain in the anterior face of the knee and loss in residual muscle strength seem to be linked to the choice of donor source. The studies are controversial in defining whether these comorbidities (such as anterior knee pain, patellofemoral symptoms, and weakness of the quadriceps muscle) are related to the graft harvest, a rehabilitation program, or the reduction in movement. However, complications such as fracture of the patella or the proximal portion of the tibia and patellar tendon rupture are clearly attributed to the process of collecting the patellar graft.^15^

As the number of primary ACL reconstructions grows, the need for revision surgery has increased significantly, and while the ideal graft choice continues to be unresolved, the contralateral patellar tendon has emerged as an option. Studies on ACL injury revision procedures and the use of this graft type found no adverse effects over the long term, and also showed a potentially more rapid post-operative recovery, with better functional scores and an even lower rate of complications.^6^ ^,^ ^9^

However, because collection of the patellar tendon graft leads to some degree of morbidity to the donor site, such as decreased sensitivity, difficulty kneeling, and quadriceps muscle weakness, orthopedic surgeons tend to choose other graft sources as their first option.^17^

In 1994, a study by Rubinstein reported the results of using contralateral patellar tendon grafts in ACL reconstruction revision procedures and proposed the use of this tendon in primary reconstruction based on the results obtained in patients related to recovery of knee range of motion, muscular strength of the quadriceps in both knees, in both the reconstructed and the donor knees. In another study in 1998, Bruck found good results for the use of this graft in ACL reconstructions, with low donor site morbidity.^5^ ^,^ ^10^

Other subsequent articles showed that the contralateral patellar tendon could be used in primary ACL reconstructions, restoring range of motion and muscle strength as early as an ipsilateral patellar tendon graft, and consequently could offer a faster full-capacity return to sports without compromising maximum stability, since after proper post-surgical rehabilitation there was no loss of strength or subjective symptoms that were not resolved.^11^ ^,^ ^17^

On the other hand, one study showed that the losses in isokinetic muscle strength after ACL revision seem similar to those observed after primary reconstruction of this ligament using the same surgical technique and grafts of the ipsilateral patellar tendon, ipsilateral ischiotibial tendons, and contralateral patellar tendon. Additionally, contralateral patellar tendon graft appeared to offer no advantages over the ipsilateral graft, because all the symptoms related to morbidity in the donor site are transferred to the healthy knee, and return to sports or professional activities is not faster.^14^ ^,^ ^16^

This present study shows that the contralateral patellar tendon graft has been used for at least twenty years, and studies are still recent. Of the 11 articles included in this review, no study used evidence level I, and studies with levels IV and V were discarded according to the exclusion criteria. Eight articles were at level II and 3 at level III. Therefore, this review demonstrates a lack of articles with a high evidence levels and A-level recommendation which compared the contralateral patellar tendon to other graft types. 

Despite the lack of these studies with higher levels of evidence, it is important to emphasize that there are cohort studies with a large number of patients, most notably by Shelbourne, who authored or co-authored 5 of the articles in this review (45.45%), all of which presented positive results for the use of contralateral patellar tendon grafts. This author also has some bibliographic reviews and texts classified as expert opinions, indicating that he is a major scholar and proponent of this type of graft. Besides these 5 articles, another 2 articles reported positive results for the use of this graft, 3 articles presented neutral results, and only 1 study indicated negative results. However, this latter case was a cohort study with evidence level II.

This literature review encountered some obstacles, such as a small number of articles addressing the use of the contralateral patellar tendon as a graft; broader descriptors were required to find more studies and then develop inclusion and exclusion criteria as well as exclusion methods involving only reading the title and abstract. 

## CONCLUSIONS

A review of studies describing the use of the contralateral patellar tendon as a graft in ACL reconstruction surgeries provides information and parameters required for decision-making. According to the literature studied, most of the articles (63.63%) presented positive results for the use of contralateral patellar tendon grafts.
